# The Relationship between Government Information Supply and Public Information Demand in the Early Stage of COVID-19 in China—An Empirical Analysis

**DOI:** 10.3390/healthcare10010077

**Published:** 2021-12-31

**Authors:** Tong Zhang, Li Yu

**Affiliations:** School of Public Affairs, Zhejiang University, Hangzhou 310058, China; yuli2020@zju.edu.cn

**Keywords:** supply–demand matching, government communication, public health emergency, government social media, crisis communication, COVID-19

## Abstract

Accurate and effective government communication is essential for public health emergencies. To optimize the effectiveness of government crisis communication, this paper puts forward an analytical perspective of supply–demand matching based on the interaction between the government and the public. We investigate the stage characteristics and the topic evolutions of both government information supply and public information demand through combined statistical analysis, text mining, text coding and cluster analysis, using empirical data from the National Health Commission’s WeChat in China. A quantitative measure reflecting the public demand for government information supply is proposed. Result indicates that the government has provided a large amount of high-intensity epidemic-related information, with six major topics being the medical team, government actions, scientific protection knowledge, epidemic situation, high-level deployment and global cooperation. The public’s greatest information needs present different characteristics at different stages, with “scientific protection knowledge”, “government actions” and “medical teams” being the most needed in the outbreak stage, the control stage and the stable stage, respectively. The subject of oversupply is “medical team”, and the subject of short supply is “epidemic dynamics” and “science knowledge”. This paper provides important theoretical and practical value for improving the effectiveness of government communication in public health crises.

## 1. Introduction

Major public emergencies are characterized by urgency, uncertainty, destructiveness and proliferation. Considered to be the most serious global public health emergency in the past 100 years, coronavirus pandemic (COVID-19) has caused more than 271.51 million confirmed cases and 5.3 million deaths (as of 15 December 2021) [1], posing severe threats to people’s daily life and social development of countries around the world.

China has experienced the unprecedented epidemic of COVID-19 in an era in which social media has fundamentally transformed information production and consumption patterns [2]. Especially when cities were under complete lockdown and citizens were restricted to staying at home, social media empowered them to address social issues [3]. There is no doubt that social media platforms facilitate the fast and easy exchange of information through sharing, discussion and communication, producing a huge amount of digital content [4]. However, it should be noted that negativity from unsubstantiated rumors can develop into extreme public reactions and cause panic during emergencies [5]. This is true especially under the circumstance that the greater the uncertainty among people, the more panic they feel [6].

In times of crisis, governmental organizations use social media to update and share information about the general public’s critical conditions [7]. Government communication helps to reduce the risk of secondary disasters [8]. Previous studies have explored the principles that the government, as an important information supplier in public emergencies, should follow in information dissemination, such as (1) information should be released centrally rather than decentralized [9]; (2)providing the information resources that the public really needs [10]; (3) improving the consistency and cooperation of information diffusion [11] and (4) clearly disseminating customized content for multiple subjects, with maximum credibility, compassion, openness and honesty [12]. In addition, empirical studies have analyzed the influence of government language styles on public participation, with present tense, informal, cognitively complex, more female, health-related and third-party related being more popular. Emotions can also lead to more participation [13]. Different stages of the crisis require different communication strategies [14]. However, there is a gap in the quantitative evaluation of the effectiveness of government communication in crisis. On the one hand, the effectiveness of government communication is difficult to evaluate [15,16,17], and there are a few studies involving the crisis. There have been empirical studies involving the public acceptance of technology [18], and social media engagement [17], but crisis communication is rarely involved. On the other hand, empirical research on crisis communication evaluation rarely uses quantitative methods. Instead, normative research and case study are more used in crisis communication evaluation [19,20]. Thereby, it is difficult to identify specific shortcomings in government communication and propose effective and accurate improvement strategies for public health crises.

Meanwhile, the public has an objective demand for timely and accurate information in public health emergencies. Literature has explored the public’s motives and needs on social media, including information search, political interests, self-development, perceived reciprocity, altruism and economic consumption [3,21]. Certain characteristics of information have been identified to attract more public attention, such as phases, emotions and topics [22]. Themes such as epidemic dynamics, popular science knowledge, policies, behavior guidance and official actions have been summarized during the epidemic [2]. Viruses, protection and economics are the most concerned [23]. In the English language, the topic with the most “likes” is economic losses, and the least is travel warnings [24]. The impact mechanism of public attention has also been explored. For example, media richness has a negative impact on public participation, and dialogue has a positive impact on public participation [25]. Public negative sentiment and rumor spread was causally interrelated [26].

Existing studies have been conducted either from the perspective of government information supply or public information demand [12,24,25]. One limitation of the above research is that it is difficult to combine the supply side with the demand side. In fact, information connects the government and the public to form a dynamic system in public crisis governance [27], which has an important impact on public satisfaction, government trust and social stability. Therefore, it is necessary to explore the relationship between government information supply and public information demand. Based on this, the effectiveness of government crisis communication can be evaluated.

Supply–demand analysis has been commonly used in research fields such as economics, management and information science. For instance, in knowledge management, the low matching degree of information supply and demand on the technology transfer platform has a negative impact on the transformation results of scientific and technological achievements. Recent studies put forward a crisis management framework based on supply and demand, in which social media is of significance [28]. However, related empirical studies are quite limited [29], and the degree of information matching between the supply and the demand remains unknown. If there was a measure that reflects the relationship between the government information supply and the public information demand, it would be helpful for the government to adjust communication strategies in a timely manner in a public health crisis.

This paper aims to explore the effectiveness of government information release in a public health emergency by introducing an analysis framework of matching supply and demand based on government-public interaction to analyze the content and characteristics of government information as well as the changes of public demand over time. We mainly focused on WeChat data posted by the National Health Commission (NHC) of China. We collected the text data of 2237 postings of the WeChat official account of the NHC from 1 January to 31 March in 2020.The main contributions are threefold. First, we propose an analytic perspective of supply–demand matching to study government crisis communication. Previous literature did not put government supply and public demand in the same analytical framework for empirical research. In comparison, this article conducts detailed statistical analysis and visual analysis based on government social media data. Second, we elaborately code the content of government communication to clearly present the characteristics of supply and demand in depth, which is more optimized than the fine-grained data processing in relevant studies. Third, a demand–supply ratio (DSR) is proposed as a quantitative measure to reflect the matching degree of information supply and public demand in government crisis communication.

## 2. Data and Methods

**Figure 1:**
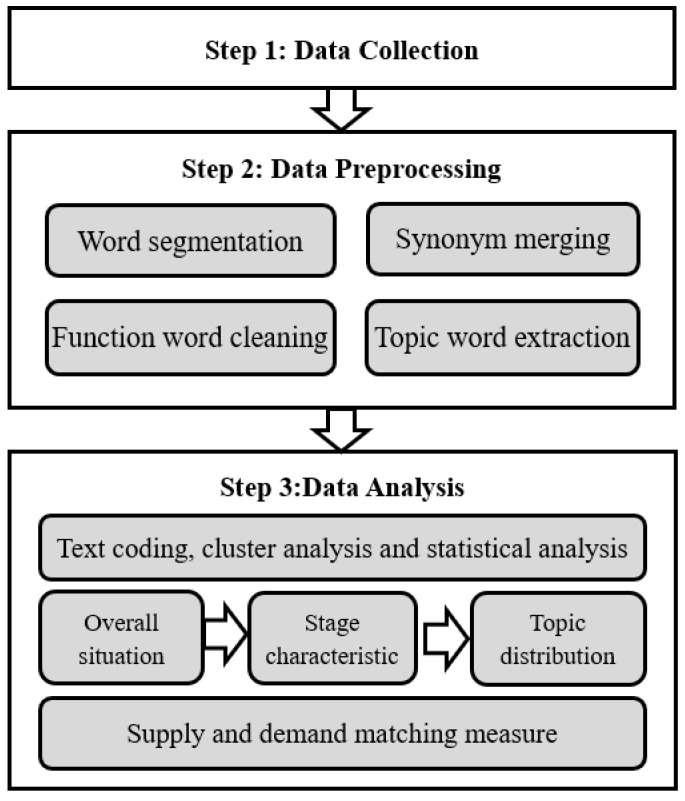
Methodological framework.

The specific data and methods (Figure 1) for analyzing the relationship of government supply and public demand are as follows:

### 2.1. Data Collection

In order to understand the relationship of information supply and information need, the data of the government WeChat official account of the National Health Commission (NHC) of China had been collected. We collected the data from GSdata (https://www.gsdata.cn/ accessed on 9 April 2020), which is a leading and representative website for social media data service in China. Specifically, we collected 2237 postings of government WeChat official account of NHC from 1 January to 31 March in 2020. The data items include tweet title, release time, number of reads, number of likes and number of comments.

The reason for choosing the data is as follows. WeChat is the most widely used social media in China, with more than 1 billion users currently. Governments at all levels and departments use WeChat official accounts to release information. NHC is the direct and professional government department that responds to public health emergencies. The information released by NHC reflects the official epidemic control plan of COVID-19, especially enabling the domestic public to obtain information for health protection. The data covers the information supplied by the government and the demand of the public.

### 2.2. Data Preprocessing

In the data preprocessing, we use text mining as the major method. Text mining method is a computer processing technology that extracts valuable information and knowledge from text data [30]. We use this method to mine and discover the thematic characteristics of the government WeChat public account postings. The details are as follows.

Specifically, we performed word segmentation, synonym merging, function word cleaning and topic word extraction on the title of the postings. The purpose of word segmentation is to divide a string of written language into words. After word segmentation, the title of each government posting becomes a series of words that can be calculated separately. Then, the text was processed by merging synonyms. Synonym merging means merging subject words with similar meanings into the same category to avoid duplication. Meanwhile, function word cleaning was carried out to delete nonsense words. It is a process of re-examining and verifying data to remove duplicate information, correct existing errors and provide data consistency. Finally, we extract topic words from the text in order to facilitate further statistical analysis.

### 2.3. Data Analysis

In data analysis, we combine the methods of text coding, cluster analysis and statistical analysis. Text coding is the basis for preliminary classification of data. Cluster analysis is based on co-occurrence network building. Statistical analysis, combined with the first two methods, runs through the whole process and is used to quantitatively analyze the government information supply and public information demand.

During the text coding process, two trained coders conducted the coding work. The first step was to establish the coding norms and construct categories as content types. After examining inter-coder reliability, the two coders started analyzing data. During the co-occurrence network building process, we used cluster analysis to draw an overall picture of government information supply and public information demand. The method is based on the co-occurrence network to cluster similar feature topics [31]. We used VOS viewer software to generate the map of the keyword co-occurrence network. The results of coding are consistent with those of co-occurrence network clustering. During the statistical analysis, quantitative measures had been applied to display the relationship of government information supply and public information demand. The details of the data analysis are presented in the next section.

## 3. Results

### 3.1. Overall Situation

In the early stage of COVID-19 in China, the NHC provided 2237 postings from 1 January to 31 March in 2020. Among them, 122 were not related to COVID-19, and 2115 were related to COVID-19. We use text coding to identify posting topics. Postings are divided into 6 categories and 27 sub-categories. The coding results are shown in Appendix A.

**Figure 2:**
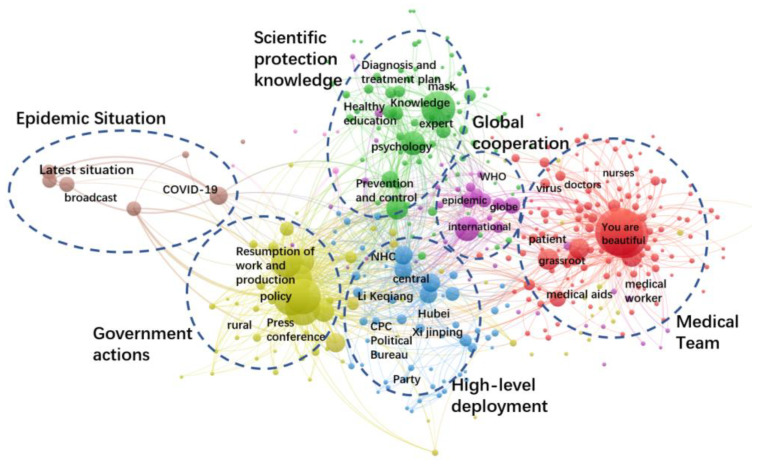
Keywords co-occurrence network of government information supply.

Figure 2 presents the network of keywords co-occurrence of the postings. There are six well-defined clusters shown in different colors, indicating the six categories of government information supply. They are: medical team, epidemic situation, government actions, high-level deployment, scientific protection knowledge and global cooperation. This is in consistent with the coding result. The node size means the number of postings for each topic.

**Figure 3:**
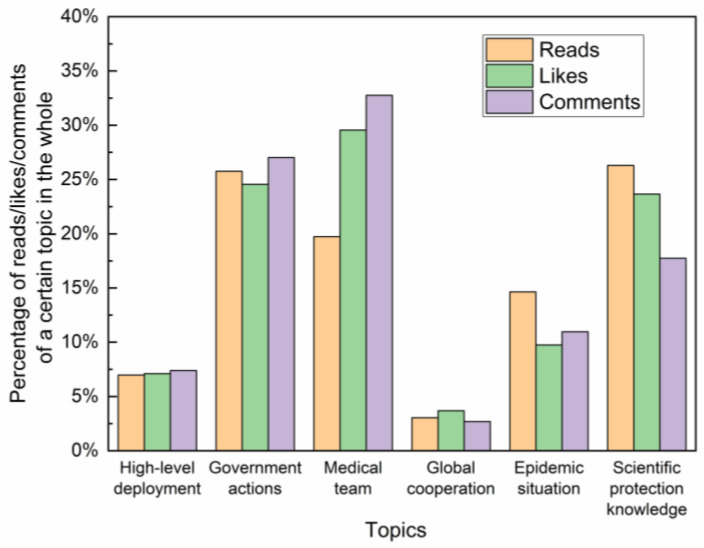
The trend of public’s attention (reads, likes and comments) in different topics.

Public information needs can be expressed through the number of reads, likes and comments in social media. We calculate the percentage of reads, likes and comments, respectively, of a certain topic in the whole in Figure 3. “Medical team”, “Government actions” and “Scientific protection knowledge” are the top three in the public’s attention. “Epidemic situation”, “High-level deployment” and “Global cooperation” are the bottom three topics in public’s attention. It can be seen that there was generally a consistent trend between reads, likes and comments. For brevity, we use the number of reads to show the public’s information demand preference in the following text.

**Figure 4:**
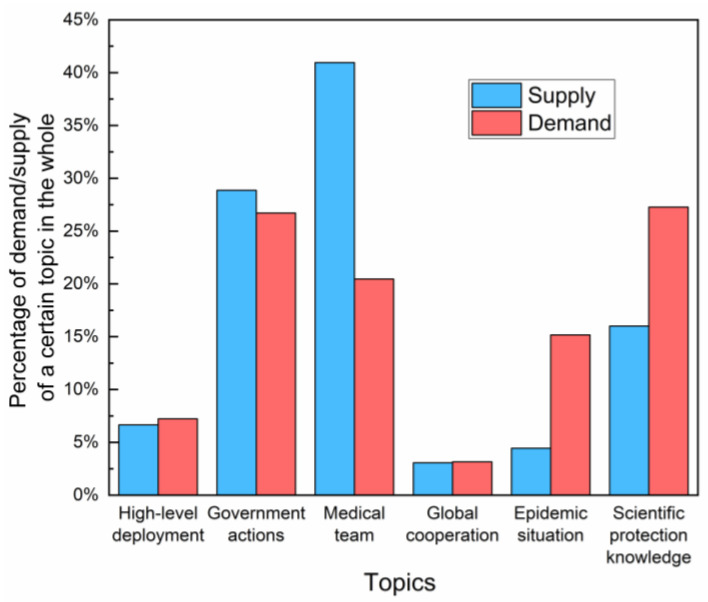
Government information supply and public information demand of different topics.

The information supply and demand of the overall topic is shown in Figure 4. By comparing the proportion of supply and demand, as is presented, there is a relative redundancy in the supply of “medical team” and a short supply of “scientific protection knowledge” and “epidemic situation”. The supply and demand almost keep in balance in the topic of “high-level deployment and “global cooperation.” That is to say, the public’s demand for the topic of “scientific protection knowledge” and “epidemic situation”, to a large extent, were greater than the government’s supply. In contrast, the public’s demand for “medical treatment” was much less than the government’s supply.

### 3.2. Stage Characteristics

To further explore the relationship between information supply and demand during emergencies, we need to analyze the stage characteristics. Previous studies on crisis management often divided emergency into three or four stages. For example, Uretsky (1991) put forward the four-stage theory of crisis communication: potential stage, outbreak stage, spread stage and resolution stage [32]. Boon-Itt and Skunkan (2020) divided the COVID-19 pandemic into three stages according to the trend of the spread and symptoms [22]. Yang et al. (2020) analyzed the data from Sina Weibo and found there exist three stages of a crisis on the whole [33]. To fully present the information supply and demand situation, in this paper, we divide the development of COVID-19 into four stages. They are: the incubation stage, the outbreak stage, the control stage and the stable stage (Table 1).

**Figure 5:**
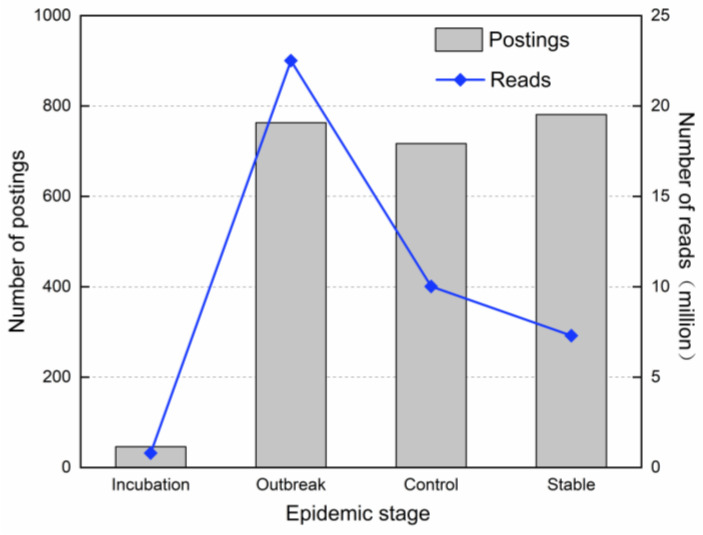
Stage characteristics of government information supply and public information demand.

During the COVID-19 period, the number of postings on the government WeChat official account reflected the central government’s emphasis on epidemic prevention and control; the number of readings reflected the attention of the public nationwide. At each stage of the epidemic’s evolution, the government’s information supply and the public’s information need have shown different characteristics. As shown in Figure 5, in terms of government information supply, from the incubation stage to the outbreak stage, the number of postings increased sharply; from the outbreak stage to the control stage, the number of postings decreased slightly and from the control stage to the stable stage, the number of postings rebounded slightly. Since the outbreak of the epidemic, the NHC has continued to release information with high intensity through the government WeChat, which reflects that the official government had conducted a high-frequency communication strategy for epidemic prevention and control.

In terms of the public’s information demand, the public’s attention was at a low level during the incubation stage. It showed a sharp increase and a peak during the outbreak stage, a significant decrease in the control stage and a further decrease in the stable stage. It exhibits that since the outbreak of the epidemic, the public has shown a high degree of attention to NHC information, reflecting a strong demand for information and a high degree of trust in the health department of the Chinese central government.

#### 3.2.1. Incubation Stage

In the incubation stage, the number of government postings was 46, but there were only 3 tweets related to COVID-19, accounting for only 6% of the total number of postings. The contents of the WeChat tweets were: “China will share the genetic sequence information of the novel coronavirus with the World Health Organization” (11 January); “Hong Kong, Macao and Taiwan expert team visits Wuhan City” (15 January) and “National Health Commission is actively carrying out the prevention and control of pneumonia caused by new coronavirus infection” (19 January).

**Figure 6:**
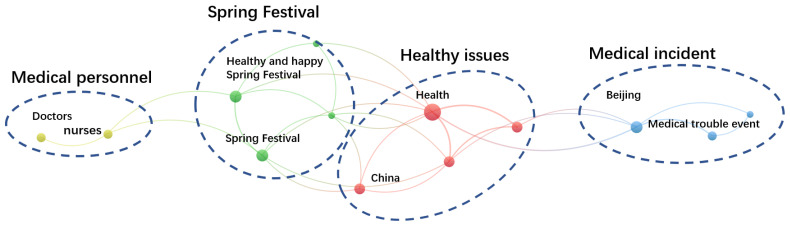
Keywords of co-occurrence network of government information supply in the incubation stage.

The keywords of the co-occurrence network of government information supply in the incubation stage is shown in Figure 6. During the incubation stage, the amount of government information provided by NHC was small and the themes were diverse. The topics covered include influenza, Spring Festival, qualification examination, medical staff, Spring Festival travel and children. The topics of government postings during the incubation stage can be summarized into four categories, namely medical personnel, spring festival, healthy issues and medical incident. However, none of them are related to COVID-19. We infer that insufficient attention was paid in the incubation stage, posing dangers to the spread of the epidemic.

Similarly, the public’s attention to information was diversified and scattered. Table 2 shows the top five tweets of WeChat public accounts read by the public. The keywords were: health conferences, medical troubles, popular science, medical staff, healthy cities, influenza, and cancer. It can be found that during the incubation stage of COVID-19, government information supply was relatively small, and public attention was at a low level. As a result, both the government and the public were negligent in prevent, which created hidden dangers for the outbreak of the epidemic.

#### 3.2.2. Outbreak Stage

**Figure 7:**
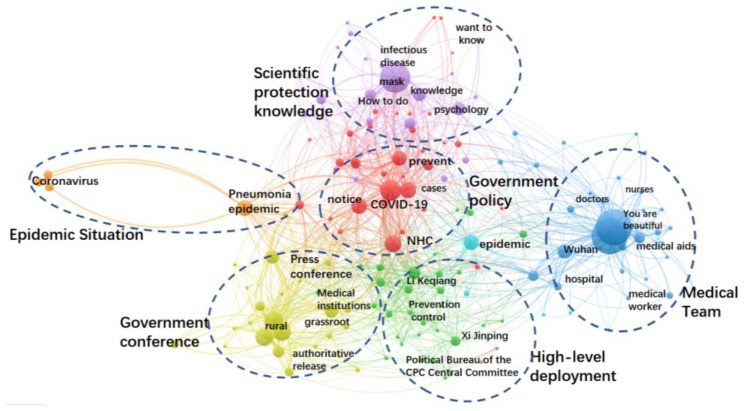
Keywords co-occurrence network of government information supply in the outbreak stage.

In the outbreak stage, the number of confirmed cases nationwide increased rapidly. At this stage, Wuhan was closed down, and the battle to defend Hubei was launched. In terms of government information supply, the total posting number of NHC was 762, with an average of nearly 30 postings per day, which was 12 times the average number of tweets in the incubation stage. Among them, 740 postings were related to COVID-19, accounting for 97% of the total release number. It can be seen that after the concentrated outbreak of COVID-19, the NHC made efforts to prevent and control the epidemic. Figure 7 shows the keywords of government information supply in the outbreak stage. There were six categories of topics, namely epidemic situation, scientific protection knowledge, government policy, medical team, high-level deployment and government conference, and the quantities of each category were similar. The figure indicates that a most comprehensive, rigorous and thorough national epidemic prevention and control was officially launched.

To further display government information supply and public information need in this stage, Table 3 listed the top 10 sub-topics with their number of postings and number of reads. “Medical worker stories” ranked 1st in terms of supply. The government hoped to record a lot of stories about medical workers, highly affirming the efforts of medical staffs, so as to boost morale for the difficult medical treatment process. In the meantime, “action guide”, “policies and measures” and “epidemic situation” got the most public attraction.

In terms of public information needs, we analyzed the postings that public paid most attention to. Specifically, there were 72 postings with more than 100,000 reads per tweet, and 421 postings with reads between 10,000 and 100,000 per tweet. Postings with more than 10,000 reads consisted of 66% of the total number of COVID-19 related postings. It can be inferred that the public maintained a high degree of attention to information related to COVID-19. Compared with the diversified themes of government information supply, the public only paid special attention to certain topics. The three topics with the highest public attention were: popular science knowledge, epidemic policy, and the latest situation of the epidemic. The representative postings that attracted the most public attention in the outbreak stage were “Announcement of the National Health Commission of the People’s Republic of China” (21 January) “, “Authoritative readings on epidemic prevention are here! Please read this guide carefully” (30 January), “Notice on printing and distributing pneumonia diagnosis and treatment plan for novel coronavirus infection (trial version 4)” (28 January), “How beautiful you are” (25 January) (songs, dedicated to the warriors in white who were doing their best to prevent and control the epidemic) and “Notice on strengthening the community prevention and control of the novel coronavirus pneumonia epidemic situation” (25 January).

#### 3.2.3. Control Stage

**Figure 8:**
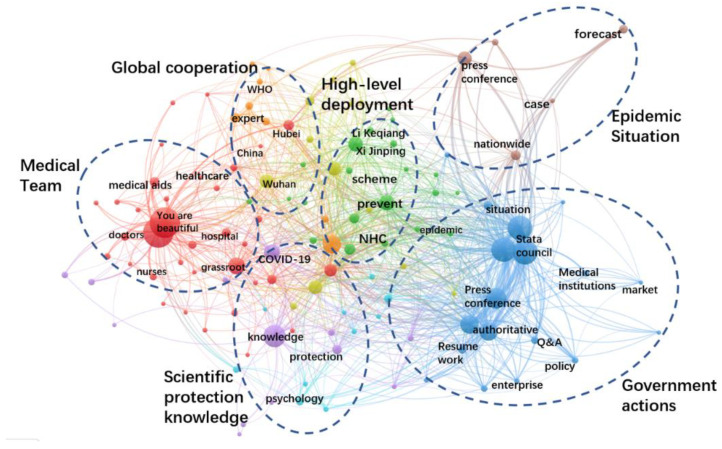
Keywords co-occurrence network of government information supply in the control stage.

In the epidemic control stage, the number of new local cases gradually dropped to single digits, and the prevention and control of the epidemic achieved important phase results. The NHC posted 717 articles, and all of them were about COVID-19. It can be seen from the intensity of the NHC’s tweeting that the Chinese government has not slackened in the prevention and control of the epidemic. In terms of quantity, the top three labels of postings were: “how beautiful you are”, “the state council joint prevention and control press conference”, and “popular science knowledge”. The keywords co-occurrence network of government information supply in the control stage is shown in Figure 8. Basically, the categories were very similar to the previous stage. A difference was that government actions accounted for a large proportion. It is worth noting that global cooperation became a new trend. This suggests that an active international exchanges and cooperation related to COVID-19 had been conducted.

Table 4 listed the top 10 sub-topics in the control stage. In terms of government supply, “medical worker stories” kept ranking the first by absolute advantage. Besides, the sub-topic won the third largest public attractions. Two new sub-topics entered the top ten list, which were “global cooperation” and “resume work and school”.

In terms of public information demand, there were 8 postings with a single reading of more than 100,000, and 262 postings with a single reading of between 10,000 and 100,000. A single posting with more than 10,000 views accounted for 38% of the total postings. At this stage, the public was still paying close attention to the epidemic information. The representative postings with high public attention were “Notice and interpretation on printing and distributing the COVID-19 diagnosis and treatment plan (trial sixth edition)” (19 February), “Guidelines for prevention and control measures for the resumption of work and production of enterprises and institutions” (22 February), “The country is set! Increasing wages for front-line medical staff, the job title evaluation is inclined! “(23 February), “China-WHO COVID-19 Joint Investigation Report was released” (29 February) and “Three departments recognize advanced collectives and individuals in the National Health System’s COVID-19 prevention and control work (attached) list)” (5 March).

#### 3.2.4. Stable Stage

**Figure 9:**
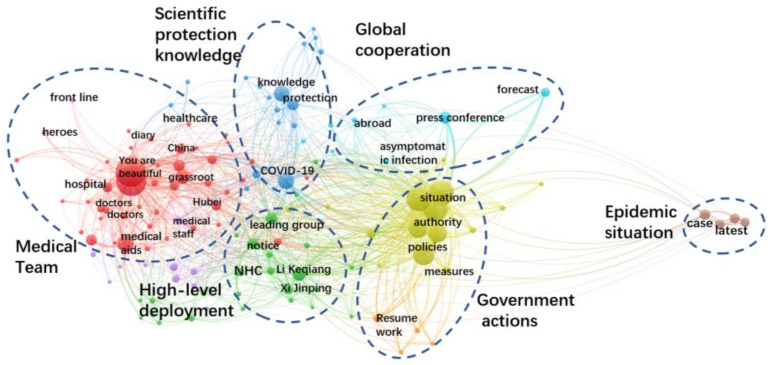
Keywords co-occurrence network of government information supply in the stable stage.

In the stable stage, sporadic cases were reported, and more infections were caused by inbound arrivals. In response to the evolving COVID-19 dynamics, the official government adopted an approach to prevent the coronavirus from entering the country and stem its domestic resurgence. The total number of NHC government postings was 781, of which 774 postings related to COVID-19, accounting for about 90%. Three topics with the highest number of postings were “How beautiful are you”, “Joint Defense and Control Conference” and “Science knowledge”. The keywords co-occurrence network of government information supply in the control stage is shown in Figure 9. Although the overall theme was similar to that of the previous stage, in terms of the amount of content, “medical team” had the highest proportion of tweets. It shows that the health sector of government was concerned about the medical team, for example, the medical workers, medical resources and substantial incentives.

Table 5 listed the top 10 sub-topics in the stable stage. “Medical worker stories” kept ranking the first, with 250 postings supplied by the government. Differently from the previous two stages, the sub-topic of “substantial medical recognition” has received widespread attention from both the government and the public in this stage.

In terms of public information needs, there were only 3 postings with a single reading of more than 100,000 and 167 postings with a single reading between 10,000 and 100,000. A single posting with a reading of more than 10,000 account for about 22% of all the postings. The representative postings of high public concern were “Attention, please be at home, 12 authoritative popular science questions and answers are here! “(11 March), “At the moment of the global pandemic, the government held a special transnational conference” (14 March), “Notice and interpretation on printing and distributing the guidelines for public scientific wearing of masks” (18 March), “Personal protection and fighting COVID-19 series of posters released” (25 March) and “Official announcement: Are asymptomatic people infected by new coronavirus contagious? Series Q&A is here! “(31 March).

### 3.3. Topic Distribution Characteristics

**Figure 10:**
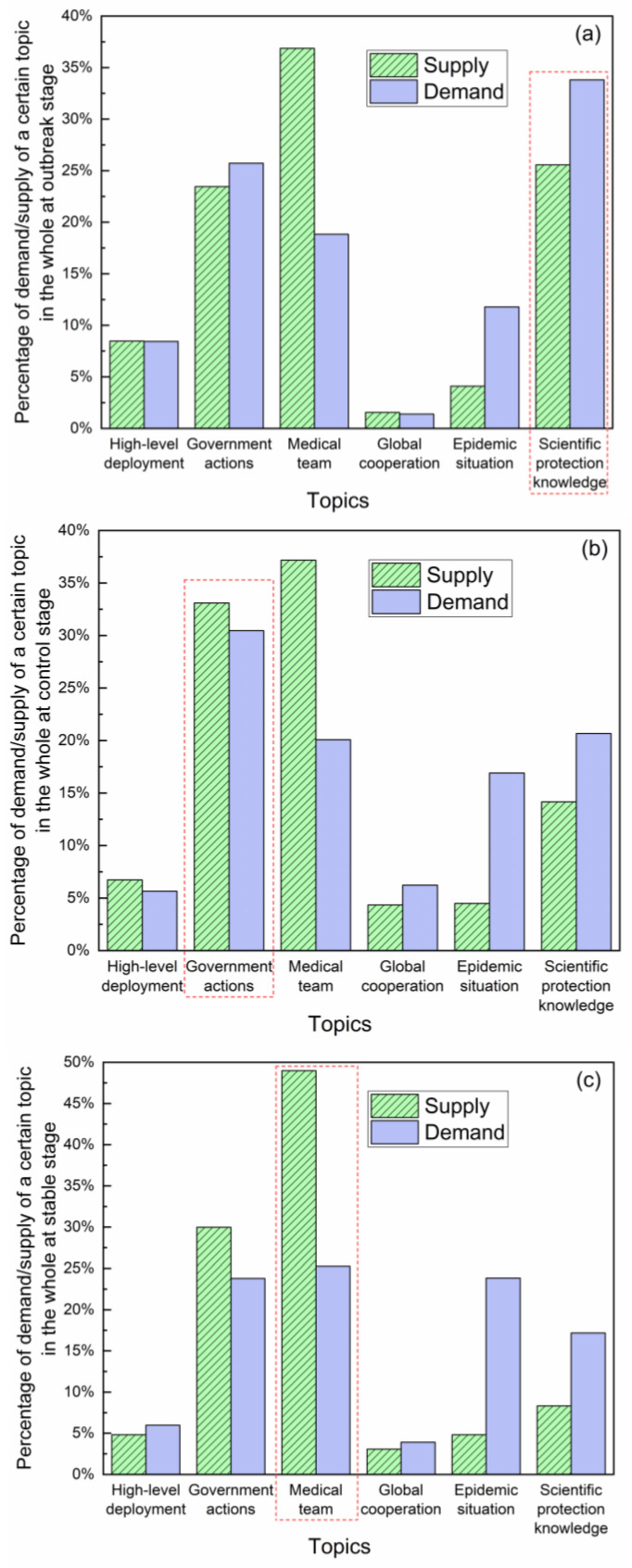
Topic distribution of government supply and public demand at (**a**) outbreak stage, (**b**) control stage and (**c**) stable stage.

To further understand the information needs of the public at different stages of the epidemic, we visually analyzed the data to present public attention on different topics at different stages. Specifically, we calculated the percentage of information supply and demand of various topics to the whole at a certain stage. Figure 10 shows the government information supply and public information demand in (a) outbreak stage, (b) control stage and (c) stable stage among the six major topic categories.

The figure reveals that “scientific protection knowledge”, “government actions” and “medical team” are the topics of highest public concerns in the outbreak stage, control stage and stable stage, respectively. Therefore, we selected these three topics that have attracted the most public attention at each stage for in-depth analysis; see Table 6 for more details.

#### 3.3.1. “Scientific Protection Knowledge” in the Outbreak Stage

**Figure 11:**
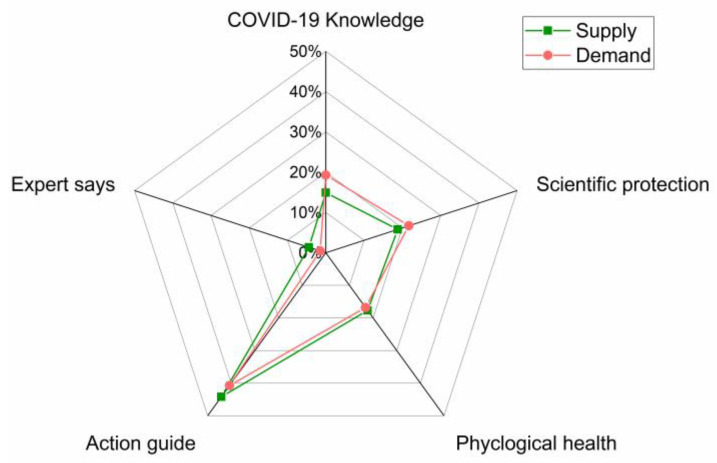
The degree of information matching between supply and demand in the outbreak stage.

In the outbreak stage, the public did not know much about COVID-19 and eagerly wanted to master relevant popular science knowledge to do better personal scientific protection. The government was an important source of authoritative information release. The “scientific protection knowledge” provided by the government can be summarized into five categories: COVID-19 knowledge, scientific protection, expert voice, phycological health, and action guide. Combining the amount of information released by the government and the amount of public reading, we have mapped the supply and the demand on various topics. As shown in Figure 11, the area covered by government supply and public demand basically overlaps. It can be seen that the government supply of various categories of subject information is basically in accordance with the public needs, showing a relevant high degree of balance.

#### 3.3.2. “Government Actions” in Control Stage

**Figure 12:**
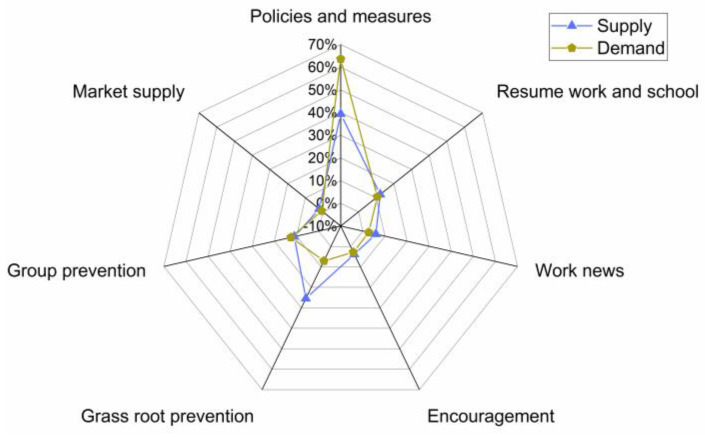
The degree of information matching between supply and demand in the control stage.

In the stage of epidemic control, “government actions” was with the highest public attention among the six major topics. The public had strong expectations for the government’s prevention and control of COVID-19. Specifically, the “government actions” were further refined and summarized into 7 categories, namely, government policies and measures, market supply, group protection, grass root prevention, encouragement, work news, and resumption of work and school. According to Figure 12, “government policies and measures” has the most supply and the greatest demand. There is a shortage of supply for “government policies and measures” and an oversupply of “grass roots prevention”. The supply and demand of other themes are basically balanced.

**Figure 13:**
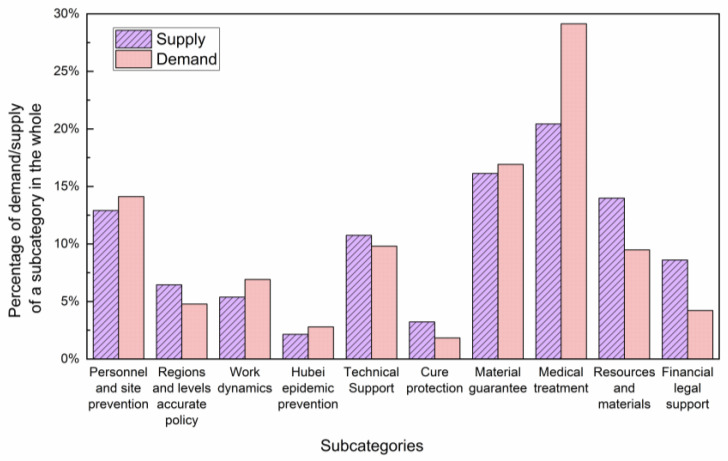
The supply and demand of subcategories of government policies and measures in the control stage.

Furthermore, we focus on the subcategories of “government policies and measures”. There are ten subcategories: personnel and site prevention, regions and levels accurate policy, work dynamics, Hubei epidemic prevention, technical support, cure protection, material guarantee, medical treatment, resources and materials and financial legal support. As shown in Figure 13, the supply and demand of each subcategory are roughly matched. The “medical treatment” is in short supply, while the supply of “resources and material” and “financial legal support” exceeds demand.

#### 3.3.3. “Medical Teams” in Stable Stage

**Figure 14:**
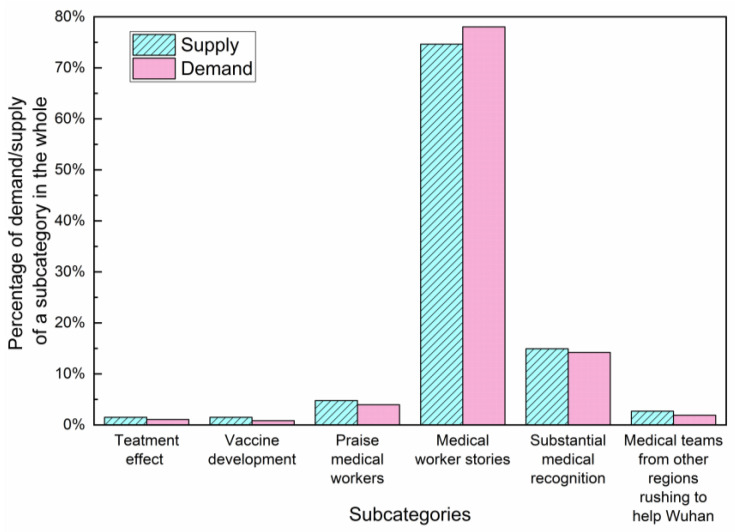
The degree of information matching between supply and demand in the stable stage.

In the stable stage, “medical teams” was the topic that public need the most (Figure 10 and Table 6). At this stage, public attention turned more to the medical aspects of the pandemic. We further studied the subcategories of “medical team” and combined specific data for visual presentation. Six subcategories have been identified in Figure 14: treatment effect, vaccine development, praise medical workers, medical worker stories, substantial medical recognition and medical teams from other regions rushing to help Wuhan. It is shown that both the supply and demand of “medical worker stories” were the largest. This indicates that a large number of medical worker stories emerged and were welcomed by the public. In the war against COVID-19, medical staff are heroes, and their touching deeds and dedication deserve public attention.

### 3.4. Measure of Demand–Supply Ratio

To better understand the relationship between government information supply and public information demand, we attempt to propose a quantitative measure. We adopt the idea of supply–demand ratio, which is often used in other disciplines. For instance, in ecological research, ecological supply–demand ratio is widely used to evaluate relevant ecosystem service [34]. In medical research, the supply-to-demand ratio for oxygen determines the formation of adenosine by the heart [35]. In economic research, a similar concept is the matching degree of efficiency, which had been highly emphasized. The mismatch between the supply and demand of online-listed rental housing restricts the operational efficiency of online rental service platforms [36]. Measuring the semantic matching efficiency of supply and demand texts can help the suppliers and demanders to retrieve information accurately [37]. Thereby, we propose a demand–supply ratio (DSR), to reflect the content matching relationship between government information supply and public information demand during a public health emergency.

DSR is the ratio between the public’s information demand for a topic and the government’s information supply for that topic. DRS is an innovative measurement, although it looks similar to the concept of engagement rate. However, there are two distinguishing differences. First, the objective of the DSR is to provide a matching degree of public demand in government information supply during a public health crisis. While the objective of engagement is to support public interaction and participation, thereby leading to better-informed government decision-making [38]. Second, the nature of the DSR is to reflect the matching relationship of topics by comparing the proportion of demand to the proportion of supply, while the nature of engagement is to involve one in public affairs or in consumer activities [39,40].

In selecting the metrics, we refer to the ideas of previous scholars in using the number of post, read, retweet, like and comment to measure the activity of social media account [41,42]. We modified the ratio of interest and consumer engagement based on Ángeles Oviedo-García et al. [43], Muñoz-Expósito et al. [44] and Calderón-Monge et al. [39]. Further, we set 1/3 as the weight for the three components of public attention for convenience, since there is a consistent trend of the number of reads, likes and comments (see Figure 3). Thus, DSR is expressed by

(1) $$\mathrm{DSR}=\frac{\frac{\mathrm{RR}}{3}+\frac{\mathrm{RL}}{3}+\frac{\mathrm{RC}}{3}}{\mathrm{RP}}$$

where

RP = Ratio of number of postings on a certain topic at a certain stage to the total number of postings

RR = Ratio of number of reads on a certain topic at a certain stage to the total number of reads

RL = Ratio of number of likes on a certain topic at a certain stage to the total number of likes

RC = Ratio of number of comments on a certain topic at a certain stage to the total number of comments

**Figure 15:**
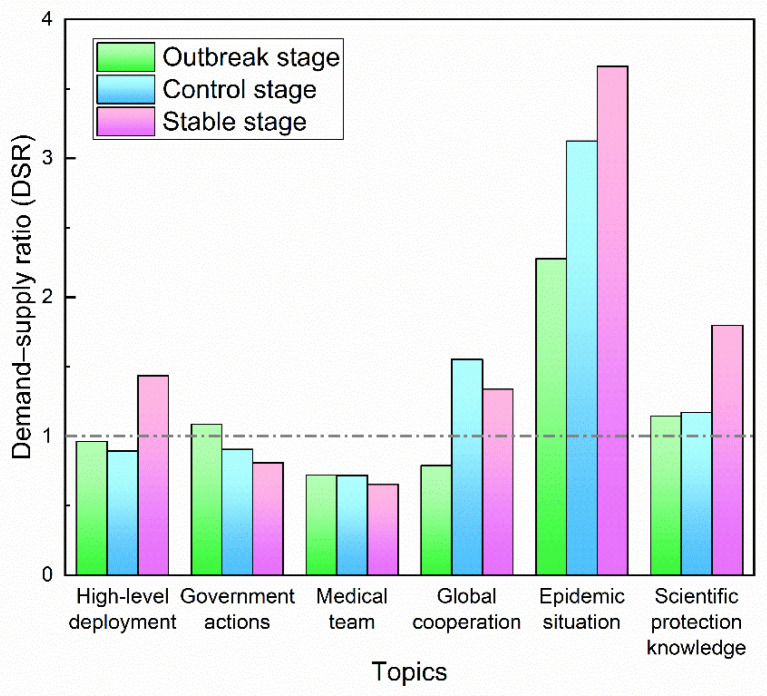
DSR on different topics at different stages.

Based on the above formula and metrics, it is clear that DSR is not judged by absolute quantity, but by the relative proportion. The measurement of public demand is the public attention ratio, that is, the proportion of a topic in the attention of all topics. The measurement of government supply is the government release ratio, namely, the proportion of a topic in the supply of all topics. Figure 15 shows the supply and demand matching degree of different themes in outbreak stage, control stage and stable stage respectively. We find that “epidemic situation” get the highest DSR in all the stages of the COVID-19. That is, the public has the most urgent need for information on the epidemic situation released by the government. Moreover, this demand level is the highest in every stage after the COVID-19 outbreak. The DSR value of “epidemic situation” is significantly higher than other topics, because the NHC posts only 1 article per day, so its overall supply is much smaller than other topics. During a pandemic, the public has deep concerns about relevant issues and fast-changing situations, expects more from the government and demands the government’s immediate attention [45]. In other words, the government might consider increasing the information supply in response to public demand. The DSR and topic distribution characteristics are displayed in Appendix B. By comparison, “scientific protection knowledge” possesses the second highest DSR in all the stages, whose overall volume is larger than “epidemic situation”, “high-level deployment” and “global cooperation”. Focusing public attention on verified information can be an ethical use of social media to avoid the spread of false information.

## 4. Discussion

Effective government communication is essential in public health crises [12]. This paper presents the staged characteristic and theme evolutions of government information supply and public information demand by putting forward an analytical perspective of government–public interactions. A quantitative measure reflecting the public demand for government information supply is proposed, filling in the gap of quantitate evaluation on the effectiveness of government communication during a public health crisis. The above analysis helps to improve the effectiveness of government communication.

In times of public health emergencies such as the COVID-19 pandemic, health agencies need communicate with the public to share time-sensitive updates, scientific information and debunk misinformation that potentially cause confusion and harm public health [46]. Our results indicate that public health sector of China had provided a large amount of high-intensity information on epidemic prevention, which basically met public information needs during the early stage of COVID-19. The six major topics supplied by the government are: medical team, government actions, scientific protection knowledge, epidemic situation, high-level deployment and global cooperation. Government communication must be highly effective and well-coordinated to provide the best available information and advice to help manage pandemics [47]. However, sometimes the government and the public had different concerns about the crisis virus [48]. In our findings, the topics of government postings during the incubation stage rarely related to COVID-19. This is consistent with previous research conclusions, since the lessons from Wuhan suggested that the accessibility and openness of information should be enhanced in advance [49]. The government supply has been highly intensive since the outbreak stage. The “medical teams” has been the most extensive topic provided by the government throughout all stages. Besides, “scientific protection knowledge” and “government actions” are also topics of heavy supply. If we put our research in a broader perspective, besides China, many countries have spread information through social media during the COVID-19 period. Although scholars had provided governments with recommendations for establishing effective health risk communication strategies, some used systems theory as a template for analyzing government communication in the United States during the COVID-19 pandemic [10], some made comparative case analysis of city agency twitter accounts for a better communication effect [9] and some drew on key findings from scholarship in multiple social science disciplines to highlight fundamental characteristics of effective governmental crisis communication [12].

Meanwhile, in public health emergencies, public attention to different types of information changes over time [33], and the hot topic keywords at each stage were slightly different [50]. Our research also confirmed these findings. Public attention was relatively scattered in incubation stage, focused on “scientific protection knowledge” in the outbreak stage, turned to “government actions” in the control stage, and finally turned to “medical teams” in the stable stage. The topics of public concern in different countries show different characteristics. For instance, in the UK, nine distinct topics were identified within the corpus of COVID-19 tweets, including mask, support, lockdown, school/work, news, reports case/deaths, retail, global pandemic and UK government, to better understand the topics of discussion and attitudes of people surrounding the pandemic [51]. In Qatar, the main topics posted by Twitter users related to the COVID-19 were: origin of the virus; its sources; its impact on people, countries and the economy and ways of mitigating the risk of infection [24]. In Japan and Korea, there was a difference in COVID-19 related topics. “COVID-19”, “Shichonji”, “Mask”, “Daegu” and “Travel” were frequently used words in Korea, while in Japan, “COVID-19”, “Mask”, “Test”, “Impact” and “China” were identified as high-frequency words. In terms of the trend, people’s interests in the economy were high in both countries [52]. The public concern was similar to our findings, that is, the overall degree of public attention shown a downward trend in the control and stable period, whereas the prevention of government increased. Meanwhile, “Resume work and school” become people’s interest. By comparison, we found a similar characteristic of government control and public demand in East Asian countries.

The study enriches the theoretical research on government crisis communication by proposing an evaluation perspective of “supply–demand” matching. The analytic perspective was validated by applying it to empirical data from the Chinese health department. Result indicates that there is a certain degree of matching between the government information supply and public information need. Meanwhile, “mismatch” also exists in specific topics such as “epidemic situation” and “medical teams”. The value of DSR provides a quantitative measure, which can quickly and intuitively show the matching relationship between supply and demand to a certain degree. Theoretically, when the value of DSR reaches 1, the supply and demand is relatively balanced, then the government could keep its supply. If the DSR value is far from 1, that means there is a “mismatch” between supply and demand. From the perspective of economics, “mismatch” means the efficiency is not maximized. In practice, according to our findings, among the government’s supply themes, there is a redundancy of “medical teams” and an insufficiency of “epidemic situations”. Therefore, it is advisable for the government to increase its supply on “epidemic situations” and decrease its supply on “medical teams” considered from an efficient perspective. However, the supply–demand relationship embodied in DSR also has limitations. It can express the public demand based on the information provided by the government, but it cannot reflect other public demand not provided by the government. Despite of that, the DSR helps to identify specific shortcomings in government communication in order to propose effective improvement strategies for public health sectors.

## 5. Conclusions

Aiming at improving the effectiveness of government crisis communication, this paper creatively put forward an analytical perspective of supply–demand matching based on the interaction between the government and the public. Empirical results from Chinese data indicate the supply of government information basically met the public demand in the early stage of fighting against COVID-19. The topics of government communication can be classified into six categories by text analysis and coding. Public information demand had been satisfied in different stages; among them, the epidemic situation was the most urgent need throughout the epidemic. Specifically, public attention was relatively scattered in the incubation stage, focused on “scientific protection knowledge” in the outbreak stage, turned to “government actions” in the control stage, and finally turned to “medical team” in the stable stage.

Some implications stem from the above, especially in terms of government crisis communication. In theory, the analytic perspective of supply–demand matching between the government and the public is enlightening, especially the measure of DSR can be used to evaluate government crisis communication in different countries. By collecting relevant data of government social media account, related research on the evaluation of government crisis communication could be carried out under this analytic framework. In practice, public health sectors can quickly and intuitively understand the relationship between government information supply and public information demand by calculating DSR value. In this way, it is feasible for government healthcare departments to timely adjust the corresponding information supply according to the public information demand at different stages in public health emergencies.

This paper has some limitations, which will become the direction of further research. Even though we have collected data from WeChat, the most widely used social media platform in China, WeChat is not the only social media used by Chinese public; in the meantime, the government uses Weibo and Tik-Tok as important communication channels. For further research, we will collect data from government social media accounts on other platforms to explore our research questions. An interesting research design might be to compare the supply–demand relationship of government and public on social media platforms with different characteristics. In addition, despite the innovative perspective of analysis, the supply–demand relationship embodied in DSR can only express the public demand based on the information provided by the government, but it cannot reflect other public demand not provided by the government. Therefore, we consider collecting data in combination with other research methods to further explore the undiscovered public information needs.

## Figures and Tables

**Table 1:** Key events and stage division of early COVID-19 in China.

Epidemic Stage	Timeline	Key Event
Incubation	1 January to 19 January	On 20 January, expert in National Health Commission (NHC) confirmed that the virus spreads from person to person.
Outbreak	20 January to 15 February	On 15 February, the press conference reported: the overall epidemic situation has become positive.
Control	16 February to 6 March	On 16 February, the government deployed the resumption of work and production.
Stable	7 March to 31 March	On 7 March, government started to establish strict policies to prevent imported COVID-19 cases from abroad.

**Table 2:** Postings with high public attention in the incubation stage (top five).

Public Focus	Representative Postings Topics	Main Content
High-level meeting	2020 National Health Work Conference held in Beijing (7 January)	The government summarized its work of 2019, studying and strengthening the construction of the health system, and deployed key tasks for 2020.
High-level deployment	The National Health Commission is actively carrying out the prevention and control of the pneumonia epidemic caused by the new coronavirus infection (19 January)	NHC established an epidemic response and handling leading group to guide and support Hubei Province and Wuhan City in carrying out case treatment, epidemic prevention and control and emergency response.
Medical incident	Sun Wenbin sentenced to death (16 January)	Outcome of the case of Sun Wenbin’s intentional homicide (medical incident)
Healthcare worker	To do popular science for everyone, the medical staff are really talented! (9 January)	The song “Wild Wolf Disco” was released for the medical care version of Dongguan, Guangdong, to make medical science popularization for everyone.
Healthcare worker	Attention, medical candidates! Register tomorrow for the 2020 National Medical Qualification Examination (8 January)	In 2020, registration matters for the medical qualification examination will be held nationwide.

**Table 3:** Public attention (reads) on different sub-topics of the government at outbreak stage of COVID-19.

Order	Sub-Topic	Number of Postings	Number of Reads
1	Medical Worker Stories	138	1,326,366
2	Policies and measures	82	2,745,852
3	Action guide	80	2,895,514
4	Medical teams from other regions rush to help Wuhan	66	712,263
5	Scientific protection	34	1,542,802
6	Phycological health	32	1,193,447
7	Epidemic situation	29	2,478,094
8	Introduction of coronavirus	27	1,371,355
9	Encouragement	23	459,726
10	Praise medical workers	23	438,501

**Table 4:** Public attention (reads) on different sub-topics of the government at control stage of COVID-19.

Order	Sub-Topic	Number of Postings	Number of Reads
1	Medical Worker Stories	218	1,069,732
2	Policies and measures	93	1,290,945
3	Grass root prevention	60	335,533
4	Action guide	33	710,056
5	Epidemic situation	32	1,677,119
6	Global cooperation	31	618,685
7	Resume work and school	29	506,190
8	Scientific protection	29	869,504
9	Group prevention	25	584,980
10	Phycological health	24	215,447

**Table 5:** Public attention (reads) on different sub-topics of the government at stable stage of COVID-19.

Order	Sub-Topic	Number of Postings	Number of Reads
1	Medical Worker Stories	250	965,182
2	Policies and measures	61	596,512
3	Grass root prevention	52	154,172
4	Substantial medical recognition	49	334,185
5	Resume work and school	33	144,871
6	Epidemic situation	33	1,570,448
7	Global cooperation	21	2,576,53
8	Group prevention	18	274,176
9	Scientific protection	16	486,822
10	Phycological health	16	193,206

**Table 6:** Public attention (reads) on different topics of the government at different stages of COVID-19.

Stage/Topic	High-Level Deployment	Government Actions	Medical Team	Global Cooperation	Epidemic Situation	Scientific Protection Knowledge
Outbreak	1,772,850	5,406,542	3,959,285	292,337	2,478,094	7,106,485
Control	560,890	3,021,249	1,991,282	618,685	1,677,119	2,049,422
Stable	393,786	1,567,892	1,666,012	257,653	1,570,448	1,132,168

**Figure A1:**
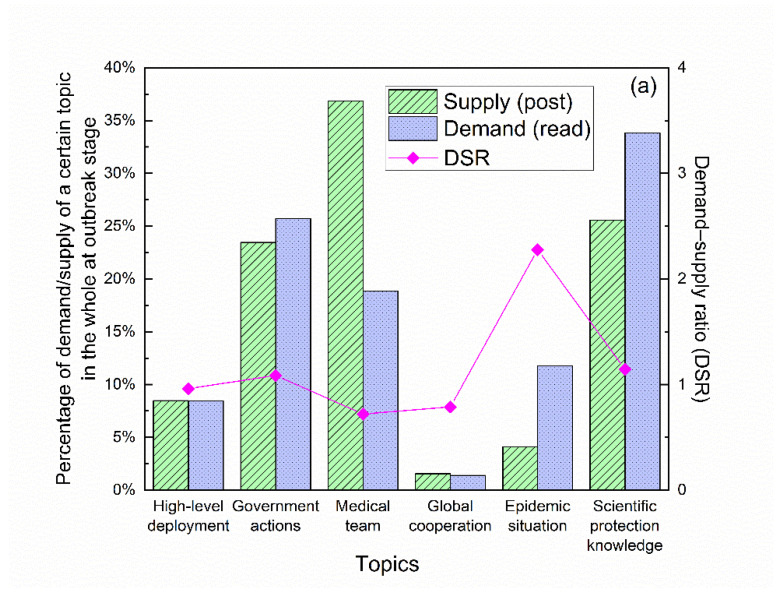

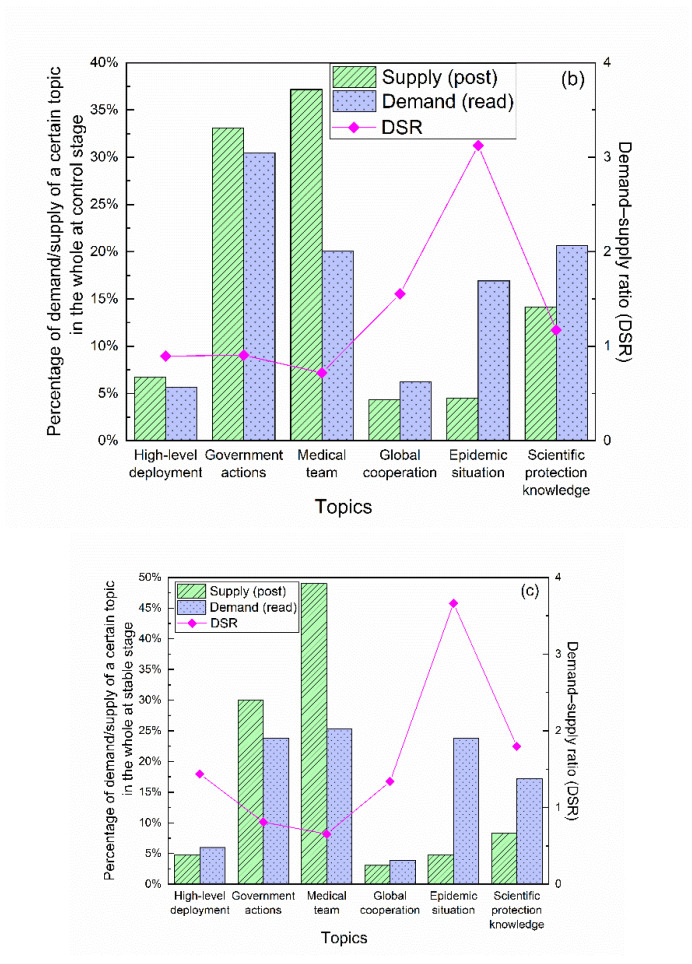
DSR and topic distribution at (**a**) outbreak stage, (**b**) control stage and (**c**) stable stage.

## Data Availability

The data used to support the findings of this study are available from the corresponding author upon request.

## Appendix A. Text Coding Results

**Table A1:** Text Coding Results of Government Postings.

Categories	Sub-Topics	Descriptions
High-level deployment	High-level deployment	Speeches and various instructions of China’s top leaders on the deployment of epidemic prevention and control.
Government actions	Policies and measures	Policies, measures and interpretations of epidemic prevention and control, including site measures, traffic measures, technical support, material support, market dynamics, etc.
	Resume work and school	Information about the resumption of work, production and schools in the middle and later stages of the epidemic.
	Work news	The NHC updated its epidemic prevention and control work, etc.
	Encouragement	Boost the morale of epidemic prevention and control through letters, speeches, songs, videos and other forms.
	Grass root prevention	Actions of urban community, rural and other grass-roots units in epidemic prevention and control, etc.
	Group prevention	Preventing suggestions for special groups such as the elderly, rural people, pregnant women and children, etc.
	Market supply	Material resources guarantee and market conditions during the epidemic period, etc.
	Professional prevention and control plan	The professional prevention and control plan was formulated and issued by the State Council of China to guide the epidemic prevention and control work of local governments, etc.
Medical team	Medical teams from other regions rush to help Wuhan	Medical teams from all over China rushed to Wuhan to provide medical assistance during the most severe period of epidemic prevention and control.
	Medical guide	Guidelines issued by various localities to facilitate people’s medical treatment.
	Safeguard measures for medical staff	Measures to protect the safety and health of front-line medical staff
	Amy’s aid	The CPC Central Military Commission sent medical staff to Wuhan for medical assistance
	Substantial medical recognition	The list of people who went to Hubei for medical assistance from all over the country, and the people on the list will be rewarded substantively.
	Medical worker stories	Touching stories of a wide variety of medical workers at all levels and places during the COVID-19
	Praise medical workers	Praise and salute the medical staff through language, painting, photography and other forms.
	Vaccine development	Vaccine and drug development in response to COVID-19.
	Epidemic judgement	Epidemic judgment released at the press conference of the joint prevention and control mechanism of the State Council.
	Diagnosis and treatment plan	Notification and interpretation of the diagnosis and treatment plan for the issuance of COVID-19
	Treatment effect	Cure and treatment effect of COVID-19.
Global cooperation	Global cooperation	Active international exchanges and cooperation on fighting COVID-19 that conducted by Chinese government.
Epidemic situation	Epidemic situation	Daily broadcast of the latest epidemic situation of COVID-19 in China.
Scientific protection knowledge	Introduction of coronavirus	Knowledge and introduction of new coronavirus.
	Scientific protection	How to carry out scientific protection? Government publishes related knowledge.
	Phycological health	Knowledge of mental health, how to overcome bad emotions, debug mental state, deal with psychological pressure, etc.
	Action guide	Appropriate behavioral guidelines for dealing with COVID-19, including personal protection guidelines for traffic behavior, home behavior, etc.
	Expert says	The voice of medical experts, such as academician Zhong Nanshan, Academician Li Lanjuan, etc.
